# Test Anxiety Levels of Board Exam Going Students in Tamil Nadu, India

**DOI:** 10.1155/2014/578323

**Published:** 2014-07-21

**Authors:** Revina Ann Mary, Gregory Marslin, Gregory Franklin, Caroline J. Sheeba

**Affiliations:** ^1^Mohamed Sathak A.J. College of Nursing, Chennai, Tamil Nadu 600 001, India; ^2^Life and Health Sciences Research Institute (ICVS), School of Health Sciences, University of Minho, 4710-057 Braga, Portugal; ^3^ICVS/3B's, PT Government Associate Laboratory, Braga, Guimarães, Portugal; ^4^Departamento de Biologia (CITAB-UM), Universidade do Minho, 4710-057 Braga, Portugal; ^5^Regenerative Medicine Program, Medicine and Biomedical Sciences Department, University of Algarve, 8005-139 Faro, Portugal; ^6^Institute for Biotechnology and Bioengineering (IBB), Centre for Molecular and Structural Biomedicine (CBME), University of Algarve, 8005-139 Faro, Portugal

## Abstract

The latest report by the National Crime Records Bureau has positioned Tamil Nadu as the Indian state with highest suicide rate. At least in part, this is happening due to exam pressure among adolescents, emphasizing the imperative need to understand the pattern of anxiety and various factors contributing to it among students. The present study was conducted to analyze the level of state anxiety among board exam attending school students in Tamil Nadu, India. A group of 100 students containing 50 boys and 50 girls from 10th and 12th grades participated in the study and their state anxiety before board exams was measured by Westside Test Anxiety Scale. We found that all board exam going students had increased level of anxiety, which was particularly higher among boys and 12th standard board exam going students. Analysis of various demographic variables showed that students from nuclear families presented higher anxiety levels compared to their desired competitive group. Overall, our results showing the prevalence of state anxiety among board exam going students in Tamil Nadu, India, support the recent attempt taken by Tamil Nadu government to improve student's academic performance in a healthier manner by appointing psychologists in all government schools.

## 1. Introduction

The educational standards of school children in India are primarily evaluated based on written examinations. Every year, the Indian government conducts two board exams, otherwise referred to as public exams, at the end of the 10th (secondary education) and 12th (higher secondary education) grades [1]. Tamil Nadu is one of the states in India and the Tamil Nadu State Board of School Examination is responsible for the 10th and 12th board examinations within the state. The scores obtained in the 10th board exams are necessary not only to get admission in good higher secondary schools but also to choose the preferred main stream of higher secondary education. Since the number of seats in high quality schools is limited compared to the number of students passing out of the 10th grade, competition to get higher secondary admission is always fierce. Students clearing up the 12th board exams leave school and enter University education. Due to guaranteed white collar job prospects, medicine, engineering, and management have been the most preferred choice of higher education by the students and/or their parents. Although there are several colleges in Tamil Nadu, only few of them are preferred by students [2], making the admission process very competitive. Thus, higher education being a prerequisite for successful future, the board exams have been the source of stress and anxiety for several students. In addition to the struggle to meet their own set values, today's students also have to satisfy the demand of their parents and the society, which adds further stress and anxiety.

Anxiety is an emotional and behavioral disorder caused by the activation of sympathetic nervous system. In the domain of education, high level of anxiety is often experienced by students during performance related activities such as, exams [3, 4]. In fact, academic examinations and school work are considered to be the most stressful events of adolescent's life [5–7]. Inefficient study, night study before exams, lack of review/revision of study materials, emotional factors, and negative/irrational thinking about exams are some of the causes of exam anxiety [8, 9]. Although some level of anxiety among students is essential to achieve success in exams [10], too much of it can have adverse effect on their performances [4]. Importantly, in students, high level of anxiety could have an impact on working memory, reasoning abilities, self-esteem, academic performance, and achievement [11, 12]. Anxiety in students can affect their physical and psychological characteristics causing panic attacks, which makes them go blank during exams, feel helpless/cold/nervous, have sweaty palms/fast breath/palpation and could even cause stomach upset [13].

Sometimes, anxiety can have extreme consequences such as tendency to attempt suicide. Unfortunately, India has one of the highest teenage suicide rates in the world [14, 15], and the number of students attempting suicide because of exam fear and pressure is increasing [16, 17]. Particularly, Tamil Nadu tops the list not only with highest suicide rates (according to the National Crime Record Bureau, 2013) [18], but also with the suicides reported due to exam failure [19]. It should be noted that suicide is an extreme manifestation of distress, suggesting that, for every reported suicide, it is likely that many mental illnesses go undiagnosed [1]. Considering the seriousness of student anxiety/stress in Tamil Nadu [20], it is imperative to systematically understand various demographic factors contributing to this state and to learn the pattern of anxiety among students. Only then, effective interventions and education reforms can be implemented to mentally prepare the students towards better academic performances. Hence, this study seeks to assess the level of state anxiety among 10th and 12th grade board exam going students in Tamil Nadu, India, and the influence of several demographic factors, namely, age, sex, class, mother tongue, religion, parent's education, living area, family type, and parent's income on student anxiety.

## 2. Methods

### 2.1. Ethics Statement

The research was conducted after the approval by the Department of Nursing, Mohamed Sathak College, Tamil Nadu, India. The aims and the procedures of the study were explained to the participants and upon their agreement; an informed written consent was signed prior to conducting the study. The study protocol and consent procedure were reviewed and approved by the Ethics Committee of Mohamed Sathak College, Tamil Nadu, India.

### 2.2. Participants

Convenient sampling technique has been chosen for this study. Sample of 100 school going students during their exam preparation period was recruited from a self-financed school in Tamil Nadu, India. Male and female students participating in the study were selected based on the following criteria: (1) age group between 15 and 18 years, (2) going to appear for 10th or 12th standard board exams in the same academic year of the survey, and (3) should be able to read and write Tamil (local language in Tamil Nadu) and English languages.

### 2.3. Procedure to Collect Demographic Data and to Measure State Anxiety Levels

Sociodemographics of the participants (e.g., age, sex, class, mother tongue, religion, parent's education, living area, family type, and parent's income) were collected through questionnaires. Three months prior to board exams, student anxiety levels were measured using the Westside Test Anxiety Scale [21], a self-reported scale to measure the temporary condition of “state anxiety” (anxiety in a specific condition). The original Westside Test Anxiety Scale instrument with 10 items was modified for the purpose of this study with 25 items, in order to adapt it for Tamil Nadu students. These 25 items enabled to measure different aspects of anxiety impairment, namely, 6 items to assess incapacity, 4 items to detect worry, 5 items to measure others view, 7 items for self-image and 3 items to estimate future security. In brief, for each item in the test anxiety inventory, the participants were asked to choose one of the 5 alternative responses such as (i) never, (ii) slight, (iii) sometimes, (iv) usually, and (v) always, which were rated on a Likert-type scale of 1 to 5 (ranging between 1 for never and 5 for always). Thus obtained scores were divided into three categories: 0 to 50 for mild, 51 to 75 for moderate, and 76 to 100 for severe anxiety levels.

### 2.4. Statistical Analysis

The statistical package for social science (IBM SPSS Statistic 20) was used to analyze the data. Independent sample *t*-test and one-way ANOVA were performed to analyze the data. 95% confidence interval was maintained. The level of anxiety was investigated based on the demographic variables; *P* value of *P* < 0.05 was considered statistically significant.

## 3. Results

### 3.1. Demographic Data of Participants

Hundred self-administered questionnaires containing 25 questions concerning sociodemographic variables were distributed among the students and all questionnaires were received back. The response rate was 100%. There were 50 male and 50 female students from diverse ethnic and socioeconomic background as indicated by the demographic data (Table 1). All participants were Indians (100%), coming from urban areas (100%), among which, most were Tamils (74%), belonging to Hindu religion (76%) and living in nuclear families (53%).

### 3.2. Level of Anxiety and Its Correlation with Demographic Variables

**Figure 1:**
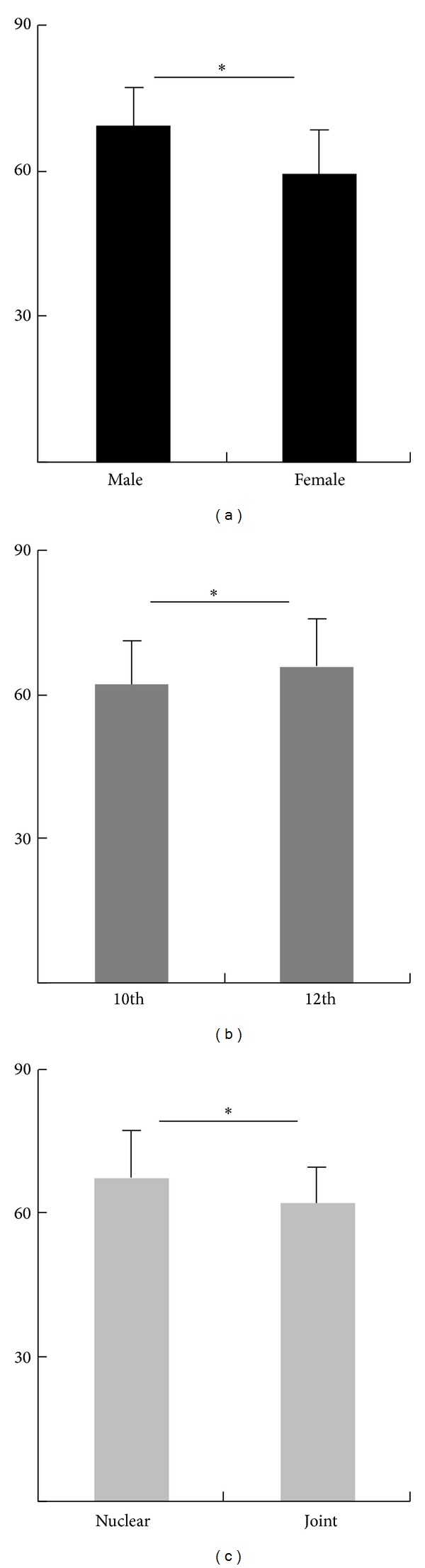
(a) Gender, (b) standard, and (c) family type.

Overall, our study showed higher anxiety levels for boys than girls, among whom 8% of boys had severe anxiety, 38% recorded moderate anxiety, and 4% had mild anxiety (Table 1). On the other hand, severe anxiety was not found among girls. The significance of difference between the mean value of male (*M* = 69.024; SD = 8.01) and female (*M* = 59.296; SD = 8.92) students was calculated by independent sample *t*-test (*t*_(98)_ = 5.736) and the existing difference was found to be statistically significant (*P* = 0.000; Figure 1(a)). Levene's test showed that the variability among males and females is more or less the same (*P* value = 0.06). So we conclude that male students had significantly higher anxiety than female students. Effect size given as a value of *r* ^2^ = 0.5 indicates a moderate practical significance (Table 2).

When the analysis was performed separately for 10th and 12th standard students, the students from 12th standard recorded 10% mild, 42% moderate, and 7% severe anxiety (Table 1). Levene's test showed that the variability among 10th and 12th is about the same (*P* value = 0.898). There is a statistically significant difference (*t*_(98)_ = −2.002; *P* = 0.048, effect size *r* ^2^ value = 0.2) between the mean value of anxiety levels between 10th (*M* = 61.85; SD = 9.3) and 12th students (*M* = 65.76; SD = 9.8) (Figure 1(b)), indicating that the 12th standard students had significantly greater anxiety than 10th students (Table 2).

Since the participants were from two different family types, namely, nuclear and joint families, we also correlated anxiety levels with the family types. Students from nuclear families showed 11% mild, 35% moderate, and 7% severe anxiety, whereas students from joint families only displayed mild and moderate anxiety of 8% and 39%, respectively (Table 1). This reveals that highest anxiety was measured in nuclear families (*M* = 66.73; SD = 10.49) compared to joint families (*M* = 61.26; SD = 8) (Figure 1(c)). Independent sample *t*-test showed statistically significant difference in anxiety levels between family types (*t*_(95.946)_ = 2.951; *P* = 0.004, effect size *r* ^2^ value = 0.3) (Table 3). Levene's test showed that the variance is significantly different among nuclear and joint families (*P* value = 0.045).

Other demographic variables such as student's age, language, religion, parent's education, and income did not show any statistical significance by having the *P* above 0.05 suggesting that there were no differences in mean anxiety levels of students belonging to different age, language, religion, parent's education, or income (Table 3).

### 3.3. Overall Anxiety Levels in 10th and 12th Grade Male and Female Students

Based on their percentage of scores the students were categorized into mild, moderate, and severe anxiety level groups with the total of 18, 74, and 8 students, respectively. The overall mean level of anxiety on Westside Test Anxiety Scale was 64.16 and the standard deviation was 9.75 (*n* = 100) (Table 4). Moreover, the total scores of the five subclasses (incapacity, worry, other's view, self-image, and future security) were assessed (Table 7) and, intriguingly, other's view contributed to high test anxiety, which was followed by future security.

The percentage of each of the 10 original Westside Test Anxiety Scale items selected by the male and female students of 10th and 12th grades was also measured (Table 5) and we found that the male students at both grades showed higher mean values than female students (Table 6). Further analysis of this date revealed that majority of the 10th standard male students showed high test anxiety in 5 items, moderate or even less anxiety in 4 items, and extremely high anxiety levels in 1 of the 10 items. In the same grade female students recorded from normal to high test anxiety between the 10 items. Table 6 clearly shows that the situation is still worse among 12th grade male students, as their anxiety levels are always ranging from moderately high to extremely high levels, whereas the anxiety levels of 12th grade female students only swing from normal to high. It should be noted that none of the items reflected extremely high anxiety levels among girls from both grades. All together these data (Table 6) support our previous observation that students from 12th grade, particularly male students experience significantly higher anxiety levels than 10th students (Table 2).

## 4. Discussion

Exam anxiety is experienced by almost every student before board exams. While mild anxiety is considered to be good for students to keep them task oriented, excess anxiety has been associated with poor performance [8]. Here, we have evaluated anxiety levels among board exam going adolescent students in Tamil Nadu, India, using a self-report semistructured questionnaire and a standardized psychological test, the Westside Test Anxiety Scale. Since high test anxiety is inversely correlated with the academic achievements and the factors influencing student performance have always been a quest for educational researchers [22], we have also correlated various demographic variables with student anxiety levels.

The results obtained from the current study show that majority of the board exam going students (74%) experience moderate level of anxiety which is in agreement with previous studies conducted among school students [23]. Pressure from school and parents, the lengthy format of Indian state government board exams (3 h), and heavy subject contents are some important factors that contribute to increased anxiety among students [1]. It is already reported that students from board exam going classes, the 10th and 12th, present significantly higher level of depression, anxiety, and stress compared to the students from 9th and 11th grade [23]. We further found that the anxiety levels manifested by 12th standard students are higher than students from 10th. This could be because 12th standard is considered as a turning point in a student's life after which they enter into university studies. In India, a student's career path for the rest of his/her life is solely determined based on the marks obtained in the 12th board exams. Thus, the fear of the future and responsibility to meet their parents/teachers expectations push the 12th standard students under tremendous stress which is reflected in the anxiety scale.

Our analysis of the influence of demographic variables, namely, sex, class, and family background, on anxiety levels shows that the level of anxiety in male students (*M* = 69.024) is higher than females (*M* = 59.296), not only when the analysis was performed using all the 25 items but also when only the original 10 items of the Westside Test Anxiety Scale were considered. This observation corroborates with the previous study conducted by Deb et al. [24] among high school students in India where they showed that adolescent boys of Kolkata city suffered from higher anxiety than adolescent girls. It is rational to think that the test anxiety and the level of exam preparation are related. Seemingly, it has been advised by the test preparation tips of University of Montana that being well prepared for the test is the best way to reduce test anxiety (http://www.umt.edu/testing/FAQ/testanxiety.php). The same has also been suggested to the students of University of Illinois (http://www.counselingcenter.illinois.edu/self-help-brochures/academic-difficulties/test-anxiety/). Unlike an underprepared student, a well-prepared student is less anxious because he/she is ready to face the questions in the exam. Here, it is worth noting that, in Tamil Nadu, the percentage of female students successfully completing 10th and 12th standards is much higher than the percentage of male students. Moreover, girls have often achieved the first five top ranks in Tamil Nadu board exams [25]. In light of this information, our results showing that male students are exposed to higher level of anxiety than female students are expectable.

Cultural practices in India might also underlie the observed higher anxiety levels among boys than girls. Even though the percentage of working women in India is steadily increasing in the recent years, till date men's income is considered as the primary financial support in majority of the families. Due to this traditional stereotyped view, parents are pressing their male children to take professional courses to improve their future job prospective and familial security, and these factors could be contributing to the observed high test anxiety among boys [24]. In fact, future security has recorded the second highest score among the measured subclasses (*M* = 3.42), after other's view (*M* = 3.55). However, female school students from another Indian state, Kerala [26], and other countries like Greece [27] and Romania [28] have recorded more anxiety levels than male students. Similarly, anxiety level was found to be higher among females than male students in universities and colleges [8, 29] and these studies have associated the night study habit of female students with the observed high anxiety levels.

As per our study, family type is another parameter that contributes to student anxiety. Among the 100 students surveyed, 53 came from nuclear families and their anxiety levels were comparatively higher than the students coming from joint families. This may be due to the fact that students from nuclear families receive less care and support from parents and relatives, because, nowadays, mostly, both parents are working to meet the highly demanding basic and social needs of the family. Previous research has associated parenting practices with anxiety [30, 31]. Parental rejection and excess control have strong implication on student anxiety [30, 32]. So, it is important that parents spend quality time with their children because this ascertains their physical and emotional availability to their children. Unfortunately, lack of time, unorganized life, and/or poor prioritization leave the children unattended and alone after school hours. Such loneliness is a major cause of stress and anxiety [1, 24], whereas, in joint families, even if the parents are unavailable, children are reared by their grandparents and/or other family members [33], which keeps them active and studious.

## 5. Study Limitation

An important limitation of this study is the size and homogeneity of the sample population. In order to derive more conclusive observations, it is recommended that this pilot study could be carried out over time, prior and after board exams and with more numbers of students from heterogeneous background such as different schools (public and self-financed schools), living area, and family conditions. Moreover, this study could also be extended to understand the effect of anxiety on student performance by treating a group of students to overcome anxiety and then comparing their exam results with the untreated control group over time. Since this is a study based on self-reports, it should be considered that the level of self-reported anxiety could be biased by the willingness of students to report their real thoughts because of gender, cultural influences, and so forth.

## 6. Conclusion

This study has shown that both 10th and 12th standard board exam going students of Tamil Nadu, India, are suffering from exam anxiety, particularly boys. Further, the influence of various demographic factors contributing to the above observed effect is analyzed and discussed. Overall, more than 70% of exam going students manifested moderate anxiety and this was particularly higher among male and 12th standard students. Our data showing the presence of high anxiety and the prevalence of moderate anxiety among adolescents suggests that it is worth providing student guidance and exam preparation tips to overcome test anxiety of 10th and 12th standard board exam going students of India, particularly Tamil Nadu, since it is one of the Indian states with high suicide rate (according to the National Crime Record Bureau, 2013) [18] associated with academic failure [19]. If required, affected students can also be directed to various antianxiety measures such as student counseling, yoga, and medications. Training programs for teachers to understand the mindset of students also enable them to ameliorate student anxiety. In fact, as per a report by one of the leading newspapers of India, The Hindu, Tamil Nadu state government has appointed psychologists in all government schools with the aim of improving the academic performance of high and higher secondary students (http://www.thehindu.com/news/cities/Coimbatore/govt-appoints-psychologists-to-counsel-students/article5260642.ece). In addition to these interventions, education reforms such as simplifying evaluation procedure could also reduce student anxiety [34]. Our results also evidence that compared to joint families students from nuclear families are prone to suffer from anxiety, suggesting the need for adequate parental care and communication between parents and adolescent children. Together, these practices are expected to help students to tackle academic stressors efficiently without compromising their performance.

## Figures and Tables

**Table 1:** Sociodemographic variables.

Demographic variables	Frequency	Percentage	Anxiety level		
			Mild	Moderate	Severe
Age					
15 years	19	19%	2	17	1
16 years	22	22%	5	16	4
17 years	42	42%	10	28	3
18 years	17	17%	1	13	—
Sex					
Male	50	50%	4	38	8
Female	50	50%	14	36	—
Standard					
10th	41	41%	8	32	1
12th	59	59%	10	42	7
Language					
Tamil	74	74%	11	58	5
Hindi	5	5%	2	3	—
Others	21	21%	5	13	3
Religion					
Hindu	76	76%	14	55	7
Christian	14	14%	2	12	—
Muslim	10	10%	2	7	1
Living area					
Urban	100	100%	18	74	8
Rural	—	—	—	—	—
Parent's education					
Primary	8	8%	6	8	5
Secondary	57	57%	12	46	3
Graduate	35	35%	—	20	—
Family type					
Nuclear	53	53%	11	35	7
Joint	47	47%	8	39	—
Parent's income					
<Rs.5000	32	32%	5	25	2
>Rs.5000	48	48%	10	35	3
>Rs.10,000	20	20%	3	14	3

0 to 50: mild, 51 to 75: moderate, and 76 to 100: severe anxieties.

**Table 2:** Independent sample *t*-test analysis for exam anxiety score by gender, class, and family type (*n* = 100).

Variables	M	SD	df	*t*	*P*	*r* ^2^	Levene's test (*P* value)
Gender							
Male	69.024	8.01	98	5.736	0.000	0.5	0.06
Female	59.296	8.92					
Standard							
10th	61.854	9.30	98	−2.002	0.048^∗^	0.2	0.898
12th	65.763	9.81					
Family type							
Nuclear	66.73	10.49	95.946	2.951	0.004^∗^	0.3	0.045
Joint	61.26	8					

M: mean; SD: standard deviation; df: degrees of freedom; *t*: independent sample *t*-test; ^∗^ *P* < 0.05 is considered as statistically significant; *r* ^2^: effect size.

**Table 3:** One-way ANOVA analysis for exam anxiety score by age, language, religion, parents' education, and income (*n* = 100).

Variables	Mean	SD	df	*F*	*P*	*r*	*η*2
Age							
15 years	61.642	7.61	3, 96	1.805	0.151	0.2	0.05
16 years	62.982	10.38					
17 years	64.076	10.66					
18 years	68.706	7.71					
Language							
Tamil	64.02	9.06	2, 97	0.251	0.779	0.07	0.005
Hindi	61.92	12.59					
Others	65.18	11.68					
Religion							
Hindu	63.41	9.74	2, 97	0.966	0.384	0.1	0.02
Christian	66.114	9.02					
Muslim	67.12	10.85					
Parent's education							
Primary	65.2	5.32	2, 97	1.149	0.321	0.1	0.02
Secondary	65.25	8.9					
Graduate	62.15	11.58					
Parent's income							
<Rs.5000	64.28	9.41	2, 97	0.869	0.422	0.1	0.02
>Rs.5000	63.1	9.98					
>Rs.10,000	66.52	9.785					

M: mean; SD: standard deviation; df: degrees of freedom; *F*: one-way ANOVA; *η*2: measure of effect size for ANOVA.

**Table 4:** Level of anxiety based on percentage of score obtained by questionnaire.

	Frequency	Mild	Moderate	Severe	Mean	SD
Overall anxiety score	100	18	74	8	64.16	9.75

0 to 50: mild, 51 to 75: moderate, and 76 to 100: severe anxieties.

**Table 5:** Westside test anxiety scale interpretation of the scores for each subclass.

Subclasses	WTAS score Mean ± SD	Interpretation
Incapacity	3.19 ± 0.46	Moderately high
Worry	3.35 ± 0.47	Moderately high
Other's view	3.55 ± 0.71	High test anxiety
Self-image	2.81 ± 0.66	High normal test anxiety
Future security	3.42 ± 0.89	Moderately high

Descriptive analysis was performed; WTAS: Westside Test Anxiety Scale; WTAS score interpretation: 1.0–1.9 comfortably low test anxiety, 2.0–2.5 normal or average test anxiety, 2.5–2.9 high normal test anxiety, 3.0–3.4 moderately high, 3.5–3.9 high test anxiety, 4.0–5.0 extremely high anxiety; scale value: 1, never, to 5, always.

**Table 6:** Percentage of each of the 10 items selected by male and female students from 10th and 12th grades.

Questions	Never				Slightly				Sometimes				Usually				Always			
	10th		12th		10th		12th		10th		12th		10th		12th		10th		12th	
	M	F	M	F	M	F	M	F	M	F	M	F	M	F	M	F	M	F	M	F
Q1	—	—	—	—	2.4	2.4	—	1.7	19.5	34.2	18.7	37.3	19.5	19.5	30.5	6.8	2.4	—	5.1	—
Q2	—	4.9	—	—	14.6	17.1	5.1	27.1	22.0	26.8	35.6	18.6	7.3	7.3	13.6	—	—	—	—	—
Q3	—	—	—	1.7	2.4	14.6	5.1	10.2	17.1	29.3	32.2	18.6	24.4	12.2	17.0	15.3	—	—	—	—
Q4	2.4	4.9	—	11.9	12.2	22.0	5.1	6.8	19.5	17.1	37.3	27.1	9.8	12.2	11.9	—	—	—	—	—
Q5	4.9	12.2	—	13.6	24.4	19.5	10.2	23.7	14.6	17.1	35.6	5.1	—	7.3	8.5	3.4	—	—	—	—
Q6	—	—	—	—	—	7.3	—	3.4	4.9	12.2	28.8	8.5	39.0	31.7	22.0	32.2	—	4.9	3.4	1.7
Q7	—	—	—	—	—	—	—	—	4.9	26.8	13.6	20.3	34.1	22.0	37.3	23.7	4.9	7.3	3.4	1.7
Q8	—	—	—	—	—	4.9	—	—	22.0	26.8	5.1	8.5	22.0	24.4	28.8	35.6	—	—	20.3	1.7
Q9	—	—	—	—	—	—	—	1.7	9.8	17.1	17.0	32.2	31.7	34.2	30.5	5.1	2.4	4.9	6.8	6.8
Q10	—	—	—	—	9.8	14.6	—	15.3	24.4	34.2	28.8	27.1	9.8	7.3	25.4	3.4	—	—	—	—

Q1: the closer I am to a major exam, the harder it is for me to concentrate on the material, Q2: when I study for my exams, I worry that I will not remember the material on the exam, Q3: during important exams, I think that I am doing awful or that I may fail, Q4: I lose focus on important exams, and I cannot remember material that I knew before the exam, Q5: I finally remember the answer to exam questions after the exam is already over, Q6: I worry so much before a major exam that I am too worn out to do my best on the exams, Q7: I feel out of sorts or not really myself when I take important exams, Q8: I find that my mind sometimes wanders when I am taking important exams, Q9: after an exam, I worry about whether I did well enough, Q10: I struggle with written assignments, or avoid doing them, because I feel that whatever I do will not be good enough. I want it to be perfect; 10th grade: 18 male and 23 female students; 12th grade: 32 male and 27 female students; F: female students; M: male students.

**Table 7:** Gender difference observed in 10th and 12th grades.

Questions	10th grade		12th grade	
	Male	Female	Male	Female
Q1	3.5 ± 0.71	3.3 ± 0.56	3.8 ± 0.6	3.1 ± 0.4^∗^
Q2	2.8 ± 0.71	2.65 ± 0.8	3.2 ± 0.6	2.4 ± 0.5^∗^
Q3	3.5 ± 0.62	2.96 ± 0.71^∗^	3.2 ± 0.6	3.04 ± 0.85
Q4	2.8 ± 0.86	2.7 ± 0.9	3.1 ± 0.6	2.3 ± 0.9^∗^
Q5	2.2 ± 0.65	2.3 ± 0.98	3.0 ± 0.6	2.0 ± 0.9^∗^
Q6	3.9 ± 0.32	3.6 ± 0.84	3.5 ± 0.6	3.7 ± 0.7
Q7	4.0 ± 0.49	3.65 ± 0.71	3.8 ± 0.5	3.6 ± 0.6
Q8	3.5 ± 0.5	3.3 ± 0.6	4.3 ± 0.6	3.6 ± 0.5^∗^
Q9	3.8 ± 0.5	3.8 ± 0.6	3.8 ± 0.6	3.4 ± 0.8^∗^
Q10	3.0 ± 0.7	2.9 ± 0.6	3.5 ± 0.5	2.7 ± 0.6^∗^

For questions Q1 to Q10 refer to Table 6; independent sample *t*-test was performed; ^*∗*^ *P* < 0.05 is considered as statistically significant; for Westside Test Anxiety Scale score interpretation refer to Table 5.

## References

[1] Rao AS (2008). Academic stress and adolescent distress: the experiences of 12th standard students in Chennai, India. *ProQuest Dissertations & Theses ProQuest*.

[2] Kumar S (2008). Engg students prefer pvt colleges. *The Times of India*.

[3] Mwamwenda TS (1994). Test anxiety and academic achievement among South African university students. *Psychological Reports*.

[4] Vitasari P, Wahab MNA, Othman A, Herawan T, Sinnadurai SK (2010). The relationship between study anxiety and academic performance among engineering students. *Procedia—Social and Behavioral Sciences*.

[5] Deinzer R, Kleineidam C, Stiller-Winkler R, Idel H, Bachg D (2000). Prolonged reduction of salivary immunoglobulin A (sIgA) after a major academic exam. *International Journal of Psychophysiology*.

[6] Lacey K, Zaharia MD, Griffiths J, Ravindran AV, Merali Z, Anisman H (2000). A prospective study of neuroendocrine and immune alterations associated with the stress of an oral academic examination among graduate students. *Psychoneuroendocrinology*.

[7] McGuire DP, Mitic W, Neumann B (1987). Perceived stress in adolescents: what normal teenagers worry about. *Canada's Mental Health*.

[8] Hashmat S, Hashmat M, Amanullah F, Aziz S (2008). Factors causing exam anxiety in medical students. *Journal of the Pakistan Medical Association*.

[9] Singh I, Jha A (2013). Anxiety, optimism and academic achievement among students of private medical and engineering colleges: a comparative study. *Journal of Educational and Developmental Psychology*.

[10] Horwitz E (2001). Language anxiety and achievement. *Annual Review of Applied Linguistics*.

[11] Mazzone L, Ducci F, Scoto MC, Passaniti E, D'Arrigo VG, Vitiello B (2007). The role of anxiety symptoms in school performance in a community sample of children and adolescents. *BMC Public Health*.

[12] McCraty R, Tomasino D, Atkinson M, Aasen P, Thurik SJ (2000). *Improving Test-Taking Skills and Academic Performance in High School Students Using HeartMath Learning Enhancement Tools*.

[13] Ruffins P A real fear: it's more than stage fright. Math anxiety can derail academic or professional success but some scholars are working to help students get over it.

[14] Geelani B Student Suicides Force Indian Government to Overhaul Education System.

[15] NDTV Suicide rates in India are highest in the 15–29 age group. http://www.ndtv.com/article/india/suicide-rates-in-india-are-highest-in-the-15-29-age-group-report-234986.

[16] Mukherji A (2008). *Around 6,000 Students Committed Suicide in 2006*.

[17] Singh SK (2012). Stress drives 40 students to suicide every year. *Deccan Herald*.

[18] Vijayakumar L, Warrier S Why Tamil Nadu tops the country in suicides. http://www.rediff.com/news/interview/why-tamil-nadu-tops-the-country-in-suicides/20131025.htm.

[19] Kumar VS (2013). *TN Tops in Suicides due to Love Failure and Exams*.

[20] Aaron R, Joseph A, Abraham S (2004). Suicides in young people in rural Southern India. *The Lancet*.

[21] Driscoll R (2007). *Westside Test Anxiety Scale Validation*.

[22] Eggen PD, Kauchak DP (1999). *Educational Psychology; Learning, Psychology of; Study and Teaching (Higher); Case Studies; United States*.

[23] Bhasin SK, Sharma R, Saini NK (2010). Depression, anxiety and stress among adolescent students belonging to affluent families: a school-based study. *Indian Journal of Pediatrics*.

[24] Deb S, Chatterjee P, Walsh K (2010). Anxiety among high school students in India: comparisons across gender, school type, social strata and perceptions of quality time with parents. *Australian Journal of Educational & Developmental Psychology*.

[25] Srinivasan R, Karpagam M Who performs better and why in higher secondary examinations?.

[26] Raakhee AS, Aparna N (2011). A study on the prevalence of anxiety disorders among higher secondary students. *Education Science and Psychology*.

[27] Lazaratou H, Anagnostopoulos DC, Vlassopoulos M (2013). Predictors and characteristics of anxiety among adolescent students: a Greek sample. *Psychiatrikē*.

[28] Robu V, Sandovici A (2012). Antecedents of baccalaureate exam anxiety: testing a model of structural links by path analysis. *Procedia—Social and Behavioral Sciences*.

[29] Armstrong KA, Khawaja NG (2002). Gender differences in anxiety: an investigation of the symptoms, cognitions, and sensitivity towards anxiety in a nonclinical population. *Behavioural and Cognitive Psychotherapy*.

[30] Rapee RM (1997). Potential role of childrearing practices in the development of anxiety and depression. *Clinical Psychology Review*.

[31] Reitman D, Asseff J (2010). Parenting practices and their relation to anxiety in young adulthood. *Journal of Anxiety Disorders*.

[32] Grüner K, Muris P, Merckelbach H (1999). The relationship between anxious rearing behaviours and anxiety disorders symptomatology in normal children. *Journal of Behavior Therapy and Experimental Psychiatry*.

[33] Bahadur A, Dhawan N (2008). Social value of parents and children in joint and nuclear families. *Journal of the Indian Academy of Applied Psychology*.

[34] Gwalani P (2012). Simplified evaluation lowers anxiety among students. *The Times of India*.

